# Inventory of Fatty Acid Desaturases in the Pennate Diatom *Phaeodactylum tricornutum*

**DOI:** 10.3390/md13031317

**Published:** 2015-03-16

**Authors:** Lina-Juana Dolch, Eric Maréchal

**Affiliations:** Laboratory of Plant and Cell Physiology/Laboratoire de Physiologie Cellulaire et Végétale, Unité mixte de recherche 5168 CNRS-CEA-Université Grenoble Alpes, Institut de Recherche en Sciences et Technologies pour le Vivant, CEA Grenoble, 17 rue des Martyrs, 38054 Grenoble Cedex 9, France; E-Mail: lina-juana.dolch@cea.fr

**Keywords:** *Arabidopsis*, phaeodactylum, diatoms, fatty acid, desaturase, lipid, EPA, front-end desaturase, polyunsaturated fatty acids

## Abstract

The diatom *Phaeodactylum* is rich in very long chain polyunsaturated fatty acids (PUFAs). Fatty acid (FA) synthesis, elongation, and desaturation have been studied in depth in plants including *Arabidopsis*, but for secondary endosymbionts the full picture remains unclear. FAs are synthesized up to a chain length of 18 carbons inside chloroplasts, where they can be incorporated into glycerolipids. They are also exported to the ER for phospho- and betaine lipid syntheses. Elongation of FAs up to 22 carbons occurs in the ER. PUFAs can be reimported into plastids to serve as precursors for glycerolipids. In both organelles, FA desaturases are present, introducing double bonds between carbon atoms and giving rise to a variety of molecular species. In addition to the four desaturases characterized in *Phaeodactylum* (FAD2, FAD6, PtD5, PtD6), we identified eight putative desaturase genes. Combining subcellular localization predictions and comparisons with desaturases from other organisms like *Arabidopsis*, we propose a scheme at the whole cell level, including features that are likely specific to secondary endosymbionts.

## 1. Introduction

Diatoms are one of the most important groups of unicellular photosynthetic protists living in oceans and fresh water, with an estimated >100,000 different species [1]. They are believed to be responsible for up to one fourth of the primary productivity [2]. The organic carbon generated by diatom photosynthesis in oceans is equivalent to that of all the terrestrial rainforests combined; however, due to the position of diatoms at the base of marine food webs, this organic matter is rapidly consumed [1].

**Figure 1:**
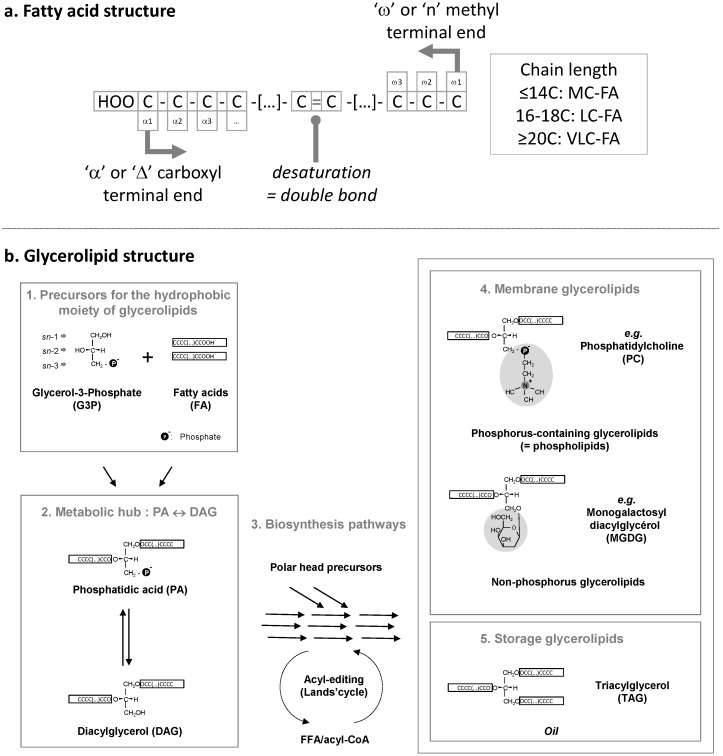
(**a**) Schematic structure of a fatty acid. Carbons are numbered either starting from the carboxyl terminal end (“α” or “Δ” nomenclature) or from the methyl terminal end (“ω” or “n” nomenclature). The chain length can vary. MC, medium chain; LC, long chain; VLC, very long chain; FA, fatty acid; (**b**) Incorporation of fatty acids in glycerolipids. Initial precursors (1), *i.e.*, glycerol-3-phosphate (G3P) and fatty acids (FA) are used to produce phosphatidic acid (PA) and its dephosphorylated form diacylglycerol (DAG), which are at the origin of all glycerolipids. Glycerolipid biosynthesis pathways (3) comprise multiple reactions leading to the production of membrane polar glycerolipids (4), or storage triacylglycerol (5). The *sn*-1, *sn*-2, and *sn*-3 numbering of the glycerol backbone is shown. This scheme gives an example of a phospholipid, phosphatidylcholine (PC), synthesized in the endoplasmic reticulum, and an example of a non-phosphorus glycolipid, monogalactosyldiacylglycerol (MGDG), synthesized in the chloroplast. It is important to note that exchanges of FAs can occur in some lipids, like PC, via a process known as acyl-editing. A PC molecule can be hydrolyzed into Lyso-PC, releasing a FA, and re-acylated using another acyl-CoA. The complete de-acylation/re-acylation process is called the Lands cycle and does not imply any net production of glycerolipid. Neo-synthesized FAs can be massively incorporated into glycerolipids at this step.

Among the organic molecules produced by diatoms, fatty acids (FAs, see general structure in Figure 1a) are essential in the nutrition of benthic and pelagic animals. FAs are precursors for three complex lipid groups, *i.e.*, glycerolipids, sphingolipids, and acylated-sterols. The most abundant acyl-lipids are glycerolipids, classified as *membrane polar glycerolipids* when one or two FAs are esterified on the glycerol backbone or *storage glycerolipids* when three fatty acids are esterified to glycerol, thus forming triacylglycerol (TAG) also known as “oil” (for review, [3]) (Figure 1b). The simplest glycerolipids synthesized *de novo* are phosphatidic acid (PA) and its dephosphorylated form diacylglycerol (DAG). PA and DAG are the precursors for all of the more complex membrane and storage glycerolipids (Figure 1b). *Non-lipid linked FAs* can serve as substrates for the production of oxygenated molecules acting as signals, called oxylipins [4]. Feeding on phytoplankton, marine arthropods and vertebrates incorporate diatom FAs into their own glycerolipids, including TAG, and thus become an important source of these FAs in human nutrition [5]. Some of the unique phytoplanktonic FAs that humans find in fish oil, including very long chain polyunsaturated fatty acids (VLC-PUFAs), cannot be provided in sufficient amounts by other food sources [5]. The specific production of VLC-PUFAs by phytoplankton has therefore attracted significant attention, and has been studied in different diatom species, in various environmental and physiological contexts.

**Figure 2:**
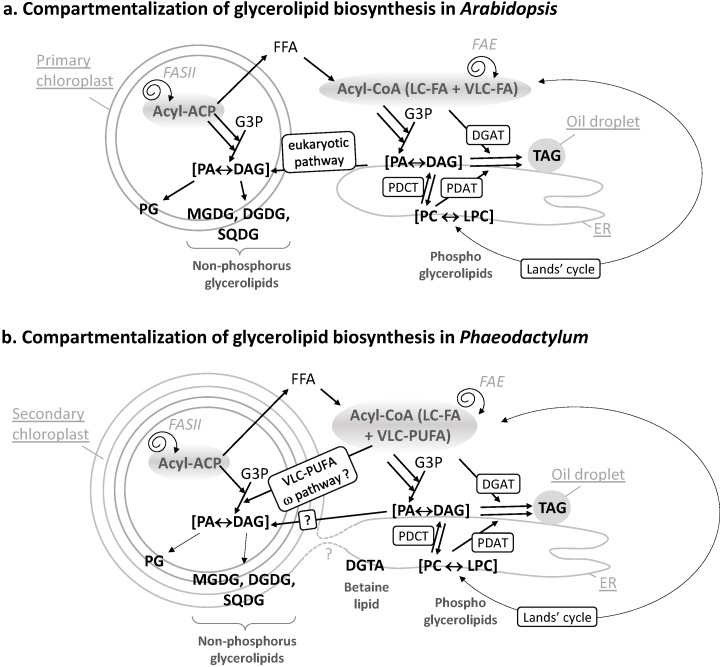
(**a**) Compartmentalization of glycerolipid biosynthesis in *Arabidopsis*. The FA synthase of type II (FASII) is located inside chloroplasts. Neo-synthesized acyl-ACP can be converted into free FAs (FFA), exported and thio-esterified to coenzyme A. Cytosolic acyl-CoA can serve for the esterification of glycerol-3-phosphate (G3P) into phosphatidic acid (PA) and its dephosphorylated form diacylglycerol (DAG). In the ER, PA, and DAG are precursors for phospholipids like phosphatidylcholine (PC). PC can undergo an acyl-editing cycle (Lands cycle), by the hydrolysis of a FA at position *sn*-2 followed by a re-acylation with an FA obtained from the acyl-CoA pool (dashed lines). In *Arabidopsis*, this process is known to incorporate more neo-synthesized fatty acids into ER glycerolipids than the stepwise acylation of G3P. Polar head exchanges can occur by the action of a PC-DAG phosphocholine transferase (PDCT), leading to the coexistence of *de novo-*synthesized or PC-derived DAG molecules with distinct FA molecular species. A third acyl can be added to DAG to form TAG, either obtained from the acyl-CoA pool by a DAG acyltransferase (DGAT) activity or by transfer from a PC molecule by a PC-DAG acyltransferase (PDAT) activity. Overall, this pathway in the ER is called the eukaryotic pathway. In chloroplasts, the prokaryotic pathway generates PA and DAG and lipids like mono and digalactosyldiacylglycerol (MGDG, DGDG), sulfoquinovosyldiacylglycerol (SQDG), or phosphatidylglycerol (PG). Some eukaryotic precursors are imported to the chloroplast; (**b**) Compartmentalization of glycerolipid biosynthesis in *Phaeodactylum*. Similar pathways are predicted to occur. Some specific features, like the import of very-long chain polyunsaturated fatty acids (VLC-PUFA) in plastid, via the omega pathway, are highlighted.

This scheme has been dissected in model organisms containing a primary chloroplast surrounded by two membranes, like the plant model *Arabidopsis thaliana* (Figure 2a) or the green alga *Chlamydomonas reinhardtii* (for review, [3,6]). The primary chloroplast derives from the engulfment of an ancestral cyanobacterium by a eukaryotic host following so-called primary endosymbiosis. The scheme is more complex in diatoms due to the presence of a chloroplast limited by four membranes, which originates from a secondary endosymbiosis [7,8]. In particular, a continuum between the ER and the outermost membrane of the plastid occurs [9] (dotted line in Figure 2b). It is not known if this connection is permanent or transient, or if lipids could transversally migrate from the ER to the outermost membrane of the chloroplast, or *vice versa*. Glycerolipid composition of each of the four membranes that surround the plastid is simply unknown. It is therefore difficult to speculate on the precise location of glycerolipid synthesis machineries and FA desaturation systems, or on the subcellular transfer of FAs within the cell. In the current state of membrane fractionation techniques, only global analyses could be performed. A reference for the glycerolipidome of *P. tricornutum* was recently detailed [10].

The inventory and subcellular localization of diatom enzymes involved in FA synthesis and modification are critical questions for advancing knowledge of this important group of oceanic biodiversity. It is also a prerequisite for the selection, domestication, or genetic engineering of diatom species. The distribution of PUFAs in glycerolipids has been studied in a few diatoms, including *Fistulifera solaris* [11], *Thalassiosira pseudonana* [12], and *Phaeodactylum tricornutum* [10]. The overall proportion of PUFAs in diatoms is tuned in response to environmental factors (for review, [13]), but their synthesis and precise biological roles are poorly understood. The analysis of FA synthesis and desaturation in diatoms is not a trivial question, mainly because it is difficult to transfer our knowledge from simple eukaryotic models, like the plant model *Arabidopsis thaliana*, to secondary endosymbionts.

When discussing FAs in photosynthetic organisms, it is usually considered that:

- medium chain FAs (MC-FAs, ≤14 carbons) and long chain FAs (LC-FAs, 16–18 carbons) are neo-synthesized in the stroma of chloroplasts;
- VLC-FAs (≥20 carbons) are generated in the ER/cytosol by secondary elongations;
- MC-FAs, LC-FAs, and VLC-FAs are incorporated in phospholipids in the endoplasmic reticulum (ER);
- MC-FAs, LC-FAs, and VLC-FAs are incorporated in non-phosphorus glycolipids in the membranes surrounding the chloroplast;
- specific FA desaturations can occur after each one of these steps.

## 2. Results and Discussion

### 2.1. Origin of Molecular Diversity of Fatty Acids: General Principles

As mentioned in the introduction, specific FA desaturations can occur after each important step in the “life” of an FA—after its *de novo* synthesis in the stroma of chloroplasts, its elongation in the cytosol, or its esterification to a glycerolipid in the ER or the chloroplast. Before listing the different desaturases of *Phaedoactylum* and their localizations, we detail therefore the general metabolic context in which they act.

FAs are carboxylic acids with an aliphatic chain of carbons, mainly even numbered, which can vary in length from 4 to >22 carbons (Figure 1a). Carbons are either numbered following the “Δ nomenclature”, starting from the α-carbon at the terminal carboxyl group (e.g., the 9th carbon following Cα = Δ9) or following the “ω nomenclature”, starting from the ω-carbon at the terminal methyl group (e.g., the 3rd carbon starting from Cω = ω3) (Figure 1a). FAs are synthesized *de novo* from acetyl-CoA and malonyl-CoA precursors through the action of enzymes called fatty acid synthases (FAS), being either a dissociated system (“FAS of type II” or FAS II) in prokaryotes and in the chloroplast, or a multi-enzymatic protein (“FAS of type I” or FAS I) in the cytosol of heterotrophic eukaryotes and some plastid-containing Chromalveolata. During the iterative process of FA synthesis, four enzymatic reactions lead to the addition of 2 carbons per cycle (for review [6]). FAs having 16 or 18 carbons are usually released by specific thioesterases (for review [6]). Some thioesterases can also release short or medium chain FAs having ≤14 carbons. Plants and diatoms both contain a FAS II system localized in the stroma of their chloroplasts (Figure 2b). VLC-FAs having ≥20 carbons are not produced by FAS, but, following a secondary addition of 2-carbon units to an acyl-CoA substrate, are catalyzed in the ER/cytosol by multi-enzymatic complexes called FA elongases (FAE). The scheme shown in Figure 2b indicates the most likely location of the different systems producing and elongating FAs in a diatom cell.

Once produced, FAs can be used as building blocks for membrane lipids, including mainly glycerolipids, but also waxes and sphingolipids, which are not discussed here. Fatty acids are then esterified to positions *sn*-1 and *sn*-2 of glycerol-3-phosphate (G3P), generating PA. PA and DAG are precursors for membrane and storage glycerolipids (Figure 1b). Based on our knowledge of *Arabidopsis* (Figure 2a), two important sites of glycerolipid production are the ER for phospholipids, mainly phosphatidylethanolamine (PE) and phosphatidylcholine (PC), and the chloroplast envelope for non-phosphorous glycoglycerolipids, *i.e.*, the sulfolipid (sulfoquinovosyldiacylglycerol, SQDG) and the galactolipids (monogalactosyldiacylglycerol, MGDG and digalactosyldiacylglycerol, DGDG) (Figure 2a). One phospholipid can be synthesized in both the ER and plastid, *i.e.*, phosphatidylglycerol (PG). When translating this scheme to diatoms, the presence of four membranes surrounding the chloroplast and the presence of a connection between the outermost membrane of the plastid and the ER, makes the localization of the phospholipid synthesis route difficult (dotted line in Figure 2a). Likewise, the precise localization of SQDG, MGDG, DGDG, and PG in the plastid cannot be predicted amongst the four membranes surrounding this organelle. One could speculate that a physical coupling of ER and chloroplast pathways might occur at the outermost plastid membrane, but this has to be demonstrated. In addition, diatoms synthesize a class of glycerolipids not found in *Arabidopsis* but synthesized in the ER of *Chlamydomonas*, a betaine lipid (BL). By contrast with *Chlamydomonas* synthesizing diacylglyceryltrimethylhomoserine (DGTS), only diacylglyceryl hydroxymethyltrimethyl-β-alanine (DGTA) could be unambiguously detected in *Phaeodactylum* [10]. Localization of lipid synthesis machineries shown in Figure 2b should therefore be confirmed experimentally and for the present article, we did not exclude any alternative possibilities.

The production of TAG was shown to be particularly complex in plants. TAG is built in the ER by addition of a FA to position *sn*-3 of a DAG, but two kinds of DAG can be used as substrate and the FA donor can be obtained from two major sources.

Concerning the origin of DAG, as mentioned above, a net incorporation of FAs into glycerolipids occurs by the stepwise esterification of G3P, generating PA and its dephosphorylated form DAG (Kennedy pathway). The acyl-CoA pool used for the stepwise acylation of G3P can either derive from plastid freshly synthesized FAs (16:0, 16:1, 18:1 molecular species) or from the de-acylation of complex lipids like PC (e.g., 18:2, 18:3 molecular species) [14] (Figure 2a, Lands cycle). A major alternative entry point of plastid neo-synthesized FAs occurs therefore by re-acylation of Lyso-PC to form PC [15,16,17,18]. Polar head exchanges can also occur by the action of a PC-DAG phosphocholine transferase (PDCT), leading to the coexistence of *de novo-*synthesized DAG or PC-derived DAG molecules with distinct FA molecular species at position *sn*-1 and *sn*-2 [19] (Figure 2a). Reverse genetics and metabolic labeling experiments have shown that acyl editing and headgroup exchange were the major mechanisms that directed polyunsaturated fatty acid flux into TAG in *Arabidopsis* [20].

The acyl added at position *sn*-3 of DAG to form TAG can be obtained from the acyl-CoA pool by a DAG acyltransferase (DGAT) activity or by transfer from a PC molecule by a PC-DAG acyltransferase (PDAT) activity [19] (Figure 2a).

In plants, the relative importance of *de novo* DAG *vs.* PC-derived DAG to form TAG differs between species, ranging from just a simple Kennedy pathway to a pathway where >90% of the FAs within the seed fluxes through PC before incorporation into TAG [19,21]. All corresponding genes have been identified in diatoms [22] and are proposed to act in TAG biosynthesis in *Phaedoactylum* (Figure 2b). In *Phaeodactylum* cells grown in a nutrient-rich medium, TAG contains mostly neo-synthesized FA molecular species, *i.e.*, 14:0/16:1/16:1 (6.5%), 14:0/16:1/16:0 (9.3%), 16:1/16:1/16:1 (11%), 16:1/16:1/16:0 (23.5%), 16:1/16:0/16:0 (16%), and 16:1/16:0/20:5 (5%), a composition that is distinct from that of PC containing high proportions of MC-PUFAs and VLC-PUFAs [10]. In low-phosphate or low-nitrogen conditions, TAG remains 16:0 and 16:1-rich [10], indicating that in *Phaeodactylum*, the production of TAG most likely relied on a DAG substrate synthesized via the Kennedy pathway and on the combined activity of a DGAT and a PDAT adding a third acyl-group at position *sn*-3.

In summary, the first source of molecular diversity of FAs lies therefore in their chain length (from 8 to 22 carbons), with:

- two distinct FA pools, an acyl-ACP pool in the stroma of the chloroplast, and one or multiple acyl-CoA pool(s) in the cytosol (possibly a LC-FA-CoA pool used for the bulk of TAG synthesis and a VLC-FA-CoA pool used for membrane phospholipids);
- two distinct FA elongation systems, a FAS II in the chloroplast, and a FAE in the ER/cytosol;
- two distinct sites of glycerolipid synthesis, phospholipid (PE, PC, *etc.*), betaine lipid (BL), and TAG pathways in the ER and a non-phosphorous glycerolipid (SQDG, MGDG, DGDG) and PG pathway in the chloroplast, with some possible connections at the level of the outermost chloroplast membrane.

### 2.2. Classification of Fatty Acid Desaturases: General Principles

The positions of unsaturations are numbered either following the “Δ” or “ω” nomenclature (Figure 1a). Desaturations are introduced by enzymes called FA desaturases. Desaturation does not occur on all possible FA substrates: desaturases operate when FAs are presented in an appropriate form, either linked to ACP, CoA, or when FAs are esterified at positions *sn*-1, *sn*-2, or *sn*-3 of the glycerol backbone in glycerolipids (Figure 1a). Double bonds are not introduced randomly but at very specific positions of FAs. All FA desaturases use a diiron cluster for catalysis [23,24,25,26]. Two main classes of FA desaturases have been identified:

- The first class corresponds to *soluble enzymes*, adding a double bond to an acyl-ACP substrate [25,26]. They exist only in the stroma of chloroplasts and their phylogenetic origin is puzzling as cyanobacteria do not have such a system [26]. They use Ferredoxin (Fd) as an electron acceptor [27].
- The second class corresponds to *transmembrane enzymes*, adding a double bond on acyl-glycerolipids, and in some cases, on Acyl-CoA substrates. Three electron acceptor systems have been characterized: Fd, for most chloroplast desaturases [27], Cytochrome b5 (Cytb5) for most ER desaturases [28], or a Cytb5-domain fused to the desaturase itself (Cytb5 fusion), in some enzymes located either in the ER [29] or in the plastid [30].

Previously characterized desaturases of *Phaeodactylum* were named either based on *Arabidopsis* homologues (like FAD2 and FAD6, [31]) or with names that do not clearly refer to a broadly accepted classification of desaturases (like PtD5 and PtD6, [32]). *Arabidopsis* being considered as a well-known reference, we proposed whenever possible some names related to this model.

### 2.3. The Arabidopsis thaliana Reference

**Figure 3:**
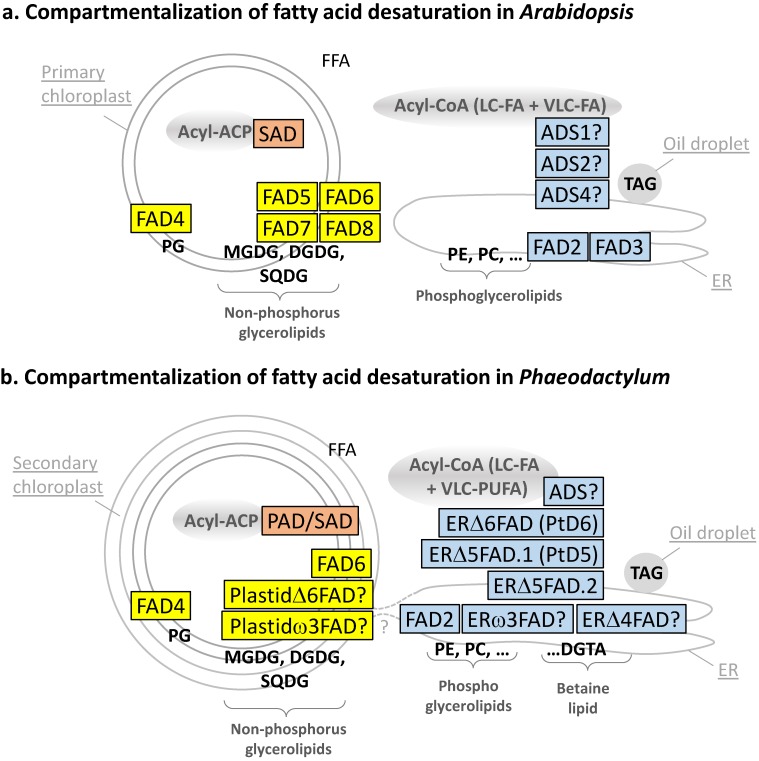
(**a**) Compartmentalization of fatty acid desaturation in *Arabidopsis*; (**b**) compartmentalization of fatty acid desaturation in *Phaeodactylum*. Enzymes in the stroma of chloroplast are shown in pink; enzymes in chloroplast membranes are shown in yellow; enzymes in endosystem membranes are shown in light blue.

Taking *A. thaliana* as a reference, the action of desaturases is dictated by the localization of the enzyme within the cell, the availability of the specific structure of the FA, whether it is linked to ACP, CoA or a class of glycerolipid, whether it has the appropriate number of carbons, and whether some desaturations are already present on the FA. Desaturations are therefore sequentially introduced, from saturated FAs (SAFAs) to monounsaturated FAs (MUFAs) and the various types of PUFAs. Table 1 summarizes the localization and substrate specificity of the major FA desaturases in *Arabidopsis*. A first observation is that similar acyl desaturations can occur in various cell compartments, catalyzed by distinct enzymes acting on different acyl lipid substrates. Both the soluble stearoyl-ACP desaturase (SAD) of the *chloroplast stroma* [25] and the membrane bound ADS1 of the *cytosol* [33,34] can generate oleic acid (18:1^Δ9^). Likewise, the *chloroplastic* FAD6 and the *cytosolic* FAD2 can produce linoleic acid (18:2^Δ9,12^), whereas the *chloroplastic* FAD7 and FAD8 and the *cytosolic* FAD3 can catalyze the production of α-linolenic acid (ALA, 18:3^Δ9,12,15^) (for review, [6]). By contrast, some desaturations can be catalyzed by a unique enzyme, like the introduction of a *cis* double bond in palmitoyl-*sn*2-MGDG by the chloroplastic FAD5 (16:1^Δ7^) [35] or the introduction of a *trans*-double bond in palmitoyl-PG by the chloroplastic FAD4 (16:*trans*-1^Δ3^) [36]. As a consequence, when we extract lipids and analyze the acyl profile of a biological sample, some acyl molecular species can be considered as signatures, like 16:3^Δ7,10,13^ for chloroplast galactolipids at position *sn*-2 or 16:*trans*-1^Δ7^ for chloroplast PG. As a corollary, if a galactolipid contains a 16:0, this acyl is at position *sn*-1, which cannot be desaturated by FAD5. Since in *Arabidopsis* MGDG is 16:3^Δ7,10,13^-rich, whereas DGDG that derives from MGDG is 16:0-rich, we can easily deduce that 16:3^Δ7,10,13^-*sn*2-MGDG is not used as a substrate for the synthesis of DGDG. As a consequence, FAD5, the committing enzyme at the origin of 16:1^Δ7^-*sn*2-MGDG, 16:2^Δ7,10^-*sn*2-MGDG and 16:3^Δ7,10,13^-*sn*2-MGDG, “locks” MGDG, preventing its conversion into DGDG [3,8]. In *Chlamydomonas*, it seems that a distinct desaturase that adds a fourth double bond on C16-MGDG also locks MGDG [30]. This Δ4-desaturase (Cr Δ4FAD) generates 16:4^Δ4,7,10,13^-*sn*2-MGDG, which cannot be used for the production of DGDG: The overexpression of Cr Δ4FAD therefore triggers the specific accumulation of MGDG [30]. In addition to SAD, ADS1, FAD2, FAD3, FAD4, FAD5, FAD6, FAD7, and FAD8 (see Table 1 and Figure 3a), *Arabidopsis* contains a set of desaturases acting on VLC-FAs, *i.e.*, ADS1, ADS2, ADS4, and probably other members of the ADS family, which are still uncharacterized [34]. The subcellular localization of *Arabidopsis* desaturases is shown in Figure 3a. Gene IDs listed in Table 1 have been used as queries to mine the *Phaedoactylum* genome (see Methods).

### 2.4. Census of Phaeodactylum Desaturases

#### 2.4.1. A Soluble Palmitoyl-ACP Δ9-Desaturase in the Stroma of Chloroplasts (16:0 → 16:1^Δ9^)

Mining the *P. tricornutum* genome, only one homologue of the *Arabidopsis* SAD gene can be found having a complete sequence and a predicted signal peptide + chloroplastic-like transit peptide (Sp + Ctp) addressing the protein to the stroma of the chloroplast: Phatr_32224. The presence of the *N*-terminal chloroplast bipartite targeting peptide was further confirmed by the detection of the Heterokont-specific ASAFAP motif using ASAFind [37] and HECTAR [38] tools (sequences shown in Supplementary Information). Since the C18 molecular species in chloroplast lipids is mainly 18:0, and the monounsaturated form of C16 is 16:1^Δ9^, and not 16:1^Δ7^ like in *Arabidopsis*, it has been proposed that the *P. tricornutum* acyl-ACP desaturase mainly acts on 16:0 and far less on 18:0, *i.e.*, acting as a palmitoyl-ACP desaturase, *i.e.*, a PAD, rather than as a stearoyl-ACP desaturase [10,31] (Table 2, Figure 3b). This soluble enzyme is likely to use Fd as its electron acceptor.

**Table 1:** Main fatty acid desaturases of *Arabidopsis thaliana*—Localization and substrate specificity.

Name	Gene ID	Subcellular Localization	Main Substrate					Main Product	
			Acyl Linked to:	Carbon Number	Presence of Double Bonds	Position and Configuration of the Introduced Double Bond	Overall Structure	Overall Structure	Name of Unsaturated FA
SAD	At2g43710 At5g16240 At3g02610 At5g16230 At5g16230 At3g02630 At1g43800	Chloroplast stroma	ACP	18	0	Δ9/*cis*	18:0-ACP	18:1^Δ9^-ACP	Oleic acid
ADS1	At1g06080	Endomembrane system	CoA	≥18	0	Δ9/*cis*	18:0-CoA	18:1^Δ9^-CoA	Oleic acid
FAD2	At3g12120	ER	Phospholipid	18	1	Δ12 (or ω6)/*cis*	18:1^Δ9^-PL	18:2^Δ9,12^-PL	Linoleic acid
FAD3	At2g29980	ER	Phospholipid	18	2	Δ15 (or ω3)/*cis*	18:2^Δ9,12^-PL	18:3^Δ9,12,15^-PL	α-Linolenic acid (ALA)
FAD5 (ADS3)	At3g15850	Chloroplast membranes	*sn*2-MGDG	16	0	Δ7 (or ω9)/*cis*	16:0-*sn*2-MGDG	16:1^Δ7^-*sn*2-MGDG	Palmitoleic acid
FAD6	At4g30950	Chloroplast membranes	*sn*1/*sn*2-MGDG/ DGDG + SQDG	16 or 18	1	ω6/*cis*	16:1^Δ7^-*sn*2-MGDG 18:1^Δ9^-*sn*1/*sn*2-MGDG/DGDG	16:2^Δ7,10^-*sn*2-MGDG; 18:2^Δ9,12^-*sn*1/*sn*2-MGDG/DGDG	7,10-Hexadecadienoic acid; Linoleic acid
FAD7/ FAD8	At3g11170 At5g05580	Chloroplast membranes	*sn*1/*sn*2-MGDG/ DGDG + SQDG	16 or 18	2	ω3/*cis*	16:2^Δ7,10^-*sn*2-MGDG 18:2^Δ9,12^-*sn*1/*sn*2-MGDG	16:3^Δ7,10,13^-*sn*2-MGDG; 18:2^Δ9,12,15^-*sn*1/*sn*2-MGDG	7,10,13-Hexadecatrienoic acid; α-Linolenic acid (ALA)
FAD4	At4g27030	Chloroplast membranes	*sn*2-PG	16	0	Δ3/*trans*	16:0-*sn*2-PG	16:1^Δ3trans^-*sn*2-PG	Δ3*-trans* Hexadecanoic acid
ADS2 ADS4 + ADS family	At2g31360 At1g06350	Endomembrane system	CoA	≥18	-	Δ9, ω6, ω7, ω9/*cis*	VLC-FA	VLC-MUFA/PUFAs	-

**Table 2:** Fatty acid desaturases of *Phaeodactylum tricornutum—*Characterized or predicted localization and substrate specificity. (*) functionally characterized; (?) based on predictions.

Name	Gene ID	Subcellular Localization	Main Substrate					Main Product
			Acyl Linked to:	Carbon Number	Presence of Double Bonds	Position and Configuration of the Introduced Double Bond	Overall Structure	Overall Structure
PAD/SAD	Phatraft_9316	Chloroplast stroma	ACP	16 and 18	0	Δ9/*cis*	16:0-ACP	16:1^Δ9^-ACP
ADS	Phatr_28797	Endomembrane system	CoA	18?	0	Δ9/*cis*?	18:0-CoA?	18:1^Δ9^-CoA?
FAD2 (*)	Phatr_25769	ER	Phospholipid/ Betaine lipid?	18	1	Δ12 (or ω6)/*cis*	18:1^Δ9^-PL (-BL?)	18:2^Δ9,12^-PL (-BL?)
ERΔ6FAD (*)	Phatr_2948	ER	Phospholipid/ Betaine lipid?	18	2	Δ6/*cis*	18:2^Δ9,12^-PL (-BL?)	18:3^Δ6,12,15^-PL (-BL?)
ERω3FAD (?)	?	ER	Phospholipid/ Betaine lipid?	18	3	Δ15 (or ω3)/*cis*	18:3^Δ6,9,12^-PL (-BL?)	18:4^Δ6,9,12,15^-PL (-BL?)
ERΔ5FAD.1 (*) ERΔ5FAD.2 (?)	Phatr_46830 Phatr_22459	ER	Phospholipid/ Betaine lipid?	20	4	Δ5/*cis*	20:4^Δ5,8,11,14,17^-PL (-BL?)	20:5^Δ5,8,11,14,17^-PL (-BL?)
ERΔ4FAD	Phatr_22510?	ER	Phospholipid/ Betaine lipid?	22	5	Δ5/*cis*	22:5^Δ7,10,13,16,19^-PL (-BL?)	22:6^Δ4,7,10,13,16,19^-PL (-BL?)
FAD6 (*)	Phatr_48423	Chloroplast membranes	*sn*1/*sn*2-MGDG/DGDG + SQDG	16	1	Δ12/*cis*	16:1^Δ9^-*sn*2-MGDG/DGDG/SQDG	16:2^Δ9,12^-*sn*1/*sn*2-MGDG/DGDG/SQDG
PlastidΔ6FAD (?)	Phatr_50443	Chloroplast membranes	*sn*2-MGDG	16	2	Δ6/*cis*	16:2^Δ9,12^-*sn*2-MGDG	16:3^Δ6,9,12^-*sn*2-MGDG
Plastidω3FAD/FAD7 (?)	Phatr_41570	Chloroplast membranes	*sn*2-MGDG?	16	3	ω3/*cis*	16:3^Δ6,9,12^-*sn*2-MGDG	16:4^Δ6,9,12,15^-*sn*2-MGDG
FAD4	Phatr_41301	Chloroplast membranes	*sn*2-PG	16	0	Δ3/*trans*	16:0-*sn*2-PG	16:1^Δ3trans^-*sn*2-PG

#### 2.4.2. Ordered Addition of Double Bonds from 16:1 to 16:2, 16:3, and 16:4

The order of addition of double bonds on the C16 backbone can simply be evaluated based on the structure of 16:1, 16:2, 16:3, and 16:4 in *Phaeodactylum* lipids. A decade ago, Domergue *et al.* showed that the FA distribution in *Phaeodactylum* was characterized by the higher abundance of 16:1^Δ9^ (25.9% of total FA), 16:2^Δ9,12^ (3.1%), and 16:3^Δ6,9,12^ (10.4%), over 16:2^Δ6,9^ (<1%) [31]. The order of the second desaturation is difficult to assess since both 16:2^Δ9,12^ and 16:2^Δ6,9^ are minor. Most unsaturated C16 FAs were detected in chloroplast lipids [10], supporting the idea that the main desaturation route is within this organelle, although some 16:1^Δ9^ desaturation into 16:2^Δ9,12^ could be partly attributed to the activity of the endosomal FAD2 enzyme, which is more specific to 18:1^Δ9^ (see below). Concerning the major chloroplast desaturation of C16 species, it might be argued on the one hand that the favorite 16:2 *substrate* for the last desaturation, generating 16:3^Δ6,9,12^, could also be the most rapidly consumed intermediate: in that case 16:2^Δ6,9^. On the other hand, one might argue that the right chloroplast 16:2 intermediate should simply be the molecular species we detect in the highest proportion: in that case 16:2^Δ9,12^. In their study of the *Phaeodactylum* chloroplast desaturase introducing a double bond at the Δ12 position, Domergue *et al.* showed that this desaturase had its highest affinity for 16:1^Δ9^ [31]. In the absence of a similar study of the desaturase introducing a double bond at the Δ6 position of C16, we thus followed the scheme proposed by these authors [31]. We also add the possible production of a 16:4^Δ6,9,12,15^ based on its detection in very low levels in MGDG [10]:

16:1^Δ9^ → 16:2^Δ9,12^ → 16:3^Δ6,9,12^ → 16:4^Δ6,9,12,15^.

#### 2.4.3. Absence of FAD5 Homologues and (16:1^Δ9^ → 16:2^Δ9,12^)-Desaturation by FAD6 in Chloroplasts

The presence of 16:1^Δ9^, and not 16:1^Δ7^, suggests that no homologue of *Arabidopsis* FAD5 would occur in *Phaeodactylum* chloroplasts. Indeed, we could not identify any putative FAD5 in the complete genome.

In chloroplast glycerolipids, C16:1^Δ9^ is the substrate for the addition of a second double bond by the action of a FAD6 homologue (corresponding to Phatr_48423), which has been characterized experimentally [31]. This enzyme was shown to act as a Δ12 desaturase, assayed after heterologous expression in the cyanobacteria *Synechococcus* [31]. The protein sequence contains a predicted Sp + Ctp *N*-terminus, containing an ASAFAP motif (Supplementary Information), consistent with its subcellular localization inside chloroplasts. The *N*-terminal Sp + Ctp of FAD6 was sufficient to target a GFP fluorescent protein into the plastid of *Phaeodactylum* [31]. FAD6 therefore produces 16:2^Δ9,12^ esterified to plastid lipids (Table 2, Figure 3B). This membrane desaturase is likely to use Fd as its electron acceptor, based on a functional study performed in a yeast heterologous system [31].

#### 2.4.4. The Question of (16:2^Δ9,12^ → 16:3^Δ6,9,12^)-Desaturation in Chloroplasts by the Action of a Cytb5-Containing PlastidΔ6FAD

The addition of a third double bond to 16:2^Δ9,12^, generating 16:2^Δ6,9,12^, cannot be deduced from the *Arabidopsis* set of desaturases, which do not harbor such activity inside chloroplasts. We identified a Cytb5-containing putative desaturase (Phatr_50443), with a Sp + Ctp *N*-terminal sequence, containing an ASAFAP motif (Supplementary Information), supporting its targeting to the chloroplast (Table 2, Figure 3b). This sequence is homologous to the Endosomal delta-6 desaturase (PtD6 or ERΔ6FAD) functionally characterized in *P. tricornutum* [32] (see below the section on front-end desaturases in the cytosol; 2.4.10) and could therefore act as a PlastidΔ6FAD enzyme. Such front-end desaturase activity has been described for a Cytb5-containing desaturase in *Chlamydomonas*, but with a Δ4 specificity [30]. Together, bioinformatic analysis of the Phatr_50443 sequence, similarity with the ERΔ6FAD enzyme of *P. tricornutum*, and analogy with *Chlamydomonas* Cytb5-containing front-end desaturase in the chloroplast support the annotation as a putative PlastidΔ6FAD (Table 2, Figure 3b, Supplementary Information). Its role in the desaturation of 16:2^Δ9,12^ into 16:2^Δ6,9,12^ should nevertheless be confirmed by functional genomic studies. This Cytb5-fusion desaturase is likely to use its own Cytb5 domain as electron acceptor.

#### 2.4.5. The Question of (16:3^Δ6,9,12^ → 16:4^Δ6,9,12,15^)-Desaturation in Chloroplasts by the Action of a Plastidω3FAD (or FAD7 Homologue)

A very low level of 16:4^Δ6,9,12,15^ could be detected in *Phaeodactylum* MGDG [10]. By mining the genome of *Phaeodactylum* we could only identify one FAD3/7/8-like sequence, which could act as a ω3 desaturase, encoded by Phatr_41570, with a predicted subcellular localization in the chloroplast, including the conserved ASAFAP motif (Supplementary Information). We called this enzyme a putative Plastidω3FAD (Table 2, Figure 3b). Future studies should be undergone to assess the precise substrate for this desaturase and whether it could act for other ω3 desaturations (see below). This membrane enzyme is likely to use Fd as an electron acceptor.

#### 2.4.6. The Specific (16:0 → 16:*trans*-1^Δ3^)-Desaturation at the *sn*-2 Position of Chloroplast Phosphatidylglycerol by FAD4

As detailed above, chloroplast phosphatidylglycerol (PG) is characterized by a 16:*trans*-1^Δ3^ at its *sn*-2, which is critical for structural and functional interactions with components of the photosynthetic machinery (for review, [39]). By mining the *Phaeodactylum* genome, a FAD4 (Table 2, Figure 3b) homologue was identified (Phatr_41301). This membrane desaturase is predicted to be localized in the chloroplast based on the prediction of a Sp + Ctp *N*-terminal sequence, containing the ASAFAP motif (Supplementary Information) and is likely to use Fd as an electron acceptor.

#### 2.4.7. The Question of Oleoyl Δ9-Desaturation in the Chloroplast and/or the Cytosol (18:0 → 18:1^Δ9^)

Small proportions of 18:1^Δ9^ FA could be detected in chloroplast lipids [10], supporting a possible production by the SAD/PAD in the stroma. Based on *Arabidopsis*, the production of 18:1^Δ9^ might be obtained via the action of the chloroplast PAD, therefore indicating some SAD activity. However, the 18:1^Δ9^-ACP thus generated in the stroma of chloroplasts should then be massively exported to the cytosol to form 18:1^Δ9^-CoA. The addition of a double bond to 18:0, forming 18:1^Δ9^, might also occur in another localization in the diatom cell, at the origin of 18:2^Δ9,12^, 18:3^Δ6,9,12^, and 18:4^Δ6,9,12,15^ [31], the substrate for VLC-FAs. Alternatively, 18:1^Δ9^-CoA might therefore be produced in the cytosol by a homologue of *Arabidopsis* ADS1 acting on 18:0-CoA. We found only one such sequence, Phatr_28797, with an *N*-terminal sequence consistent with a cytosolic localization (Table 2, Figure 3b, Supplementary Information). The role of this ADS enzyme as a putative Δ9-oleyl desaturase should be experimentally determined. This membrane desaturase is likely to use Cytb5 as its electron acceptor.

#### 2.4.8. Ordered Addition of Double Bonds from 18:1 to 18:2, 18:3, and 18:4

The main C18 unsaturated structures determined in *Phaeodactylum* were 18:1^Δ9^ (2.4% of total FA), 18:2^Δ9,12^ (2%), and very low proportions of 18:3^Δ6,9,12^ (<1%) and 18:3^Δ9,12,15^ (<1%) [31]. This indicates that the introduction of the second double bond is at position Δ12. For the following desaturations, a 3-h metabolic labeling with (^14^C) 18:0 led to the accumulation of 45.6% 18:0, 15% 18:1^Δ9^, 21% 18:2^Δ9,12^, 2.8% 18:3^Δ9,12,15^, 2.5% 18:3^Δ6,9,12^, and 0.5% 18:4^Δ6,9,12,15^ [40]. Following 18:2^Δ9,12^, it seems therefore that desaturases can operate without order, ending up with the production of 18:3 and 14 molecular species that do not accumulate, simply because they are elongated into C20 molecular species. These C20 species are very rapidly desaturated into eicopentaenoic acid (EPA): 20:5^Δ5,8,11,14,17^. Amongst all combinations, a most active route was then proposed based on metabolic labeling experiments [41,42]:

18:0 → 18:1^Δ9^ → 18:2^Δ9,12^ → 18:3^Δ6,9,12^ → 18:4^Δ6,9,12,15^.

Based on the substrate specificity of desaturases, an alternative route is also nearly as important [32,41]:

18:0 → 18:1^Δ9^ → 18:2^Δ9,12^ → 18:3^Δ9,12,15^ → 18:4^Δ6,9,12,15^.

In the next sections, we describe desaturases following the first route.

#### 2.4.9. The (18:1^Δ9^ → 18:2^Δ9,12^)-Desaturation by FAD2 in the ER

The ER FAD2 (corresponding to Phatr_25769) of *Phaeodactylum* has been finely characterized functionally in yeast heterologous system [31]. *In vitro*, this enzyme was shown to accept various substrates, like 16:1^Δ9^, 17:1^Δ9^, 18:1^Δ9^, or 20:1^Δ11^, and in all cases it added a second double bond at the level of the third carbon counted from the methyl end, generating 16:1^Δ9,12^, 17:1^Δ9,12^, 18:1^Δ9,12^, or 20:1^Δ11,14^, respectively [31]. It is not therefore a strict Δ12 or ω6 desaturase, although it is labeled as such in Table 2. The fusion of the *N*-terminal sequence of *Phaedoactylum* FAD2 to GFP led to cytoplasmic fluorescence [31] (Table 2, Figure 3b). The localization of this membrane desaturase is not completely characterized; however, it is most likely located at the level of the ER, although other membrane compartments of the endomembrane system or even the outermost membrane of the chloroplast cannot be excluded. Functional analyses in heterologous systems have shown that Cytb5 was the electron acceptor [31].

#### 2.4.10. The (18:2^Δ9,12^ → 18:3^Δ6,9,12^ and 18:3^Δ9,12,15^ → 18:3^Δ6,9,12,15^)-Desaturation by a Δ6 Front-End Desaturase, ERΔ6FAD (PtD6)

The addition of a double bond between the pre-existing ones and the carboxyl end of polyunsaturated FA is not a common process. It is catalyzed by so called front-end desaturases, which share multiple structural features, including a Cytb5 domain fused to their terminal end. The identification of the enzyme catalyzing the desaturation of 18:2^Δ9,12^ into 18:3^Δ6,9,12^ could not be deduced based on homology searches with an *Arabidopsis* template sequence. By analyzing *Phaeodactylum* genomic sequences that could encode desaturases, and by comparison with Δ6 desaturase sequences of other organisms, only one Δ6 front-end Cytb5 fusion desaturase could be identified (Phatr_2948), called here ERΔ6FAD. This desaturase corresponds to PtD6 previously characterized by Domergue *et al.* [32]. This enzyme is most likely associated to the ER or a compartment of the endomembrane system (Table 2, Figure 3b). It uses its Cytb5 domain as an electron acceptor. ERΔ6FAD was characterized functionally in heterologous systems and was shown to act equally on 18:2^Δ9,12^ and 18:3^Δ9,12,15^, generating 18:3^Δ6,9,12^ and 18:4^Δ6,9,12,15^, respectively [32].

#### 2.4.11. The Question of the (18:2^Δ9,12^ → 18:3^Δ6,9,15^ and 18:3^Δ6,9,12^ → 18:4^Δ6,9,12,15^)-Desaturation by a ERω3FAD

To our knowledge, no gene candidate has been previously identified to code for an enzyme catalyzing the addition of a double bond at position Δ15/ω3 of C18 in the ER of *Phaeodactylum*, tentatively called ERω3FAD in this article. It has been shown in plants that FAD3 enzymes are ω3 desaturases capable of adding double bonds on a variety of C18 or C20 substrates [43]. As mentioned above, we could only identify one FAD3/7/8-like sequence (Phatr_41570) that could act as a ω3 desaturase, *i.e.*, a putative Plastidω3FAD (Table 2, Figure 3b). Functional genomic studies and enzymatic assays should therefore be performed to assess whether this enzyme could act in 18:4 synthesis in *Phaeodactylum*. There might be a dual targeting of the ω3FAD gene leading to a localization of a cytosolic ERω3FAD acting on C18 substrates and a Plastidω3FAD acting on C16 substrates, and this hypothesis should be evaluated. It is also unclear whether an ω3FAD could act on both 18:2^Δ9,12^ and 18:3^Δ6,9,12^ with similar affinities, producing 18:2^Δ9,12,15^ and 18:4^Δ6,9,12,15^, respectively. The actual enzyme and its localization should be unraveled.

Regardless of the order, the actions of ERΔ6FAD and ω3FAD appear to lead to the production of an 18:4 intermediate, which does not accumulate since it is extremely rapidly elongated by a Δ6 elongase, into a C20-FA. This latter fatty acid is found in a very low proportion (<1%) [31], due to it being instantly converted into eicopentaenoic acid.

#### 2.4.12. The (20:4^Δ8,11,14,17^→ 20:5^Δ5,8,11,14,17^)-Desaturation by a Δ5 Front-End Desaturase, ERΔ5FAD.1 (PtD5) and ERΔ5FAD.2

Among the possible routes producing 20:5^Δ5,8,11,14,17^ in *Phaeodactylum*, pulse chase experiments with (^14^C)18:1^Δ9^ and (^14^C)18:2^Δ9,12^ suggested the most active one involved the elongation of 20:4^Δ8,11,14,17^ [42]. A Δ5 front-end desaturase, called here ERΔ5FAD.1 (previously described as PtD5, corresponding to Phatr_46830) was identified together with the ERΔ6FAD (PtD6) described above [32]. By testing a subset of possible 20-carbon substrates, ERΔ5FAD.1 was shown to act on 20:1^Δ11^, 20:2^Δ11,14^, 20:3^Δ11,14,17^, or 20:3^Δ8,11,14^ [32]. In these experiments, 20:3^Δ8,11,14^ was the favorite substrate, indicating that this enzyme was versatile enough to accommodate various substrates, with a very high efficiency toward the production of EPA [32]. This membrane desaturase is localized in the ER or another compartment of the endomembrane system. Like ERΔ6FAD, it uses its Cytb5 domain as an electron acceptor. It is also likely to be closely associated with components of the elongase that generates 20:4 from 18:4 [32]. By mining the genome of *Phaeodactylum*, we identified a close homologue, called here ERΔ5FAD.2 (Phatr_22459), which might act as a Δ5 desaturase as well, either as a redundant enzyme or for a specific purpose (Table 2, Figure 3b). The presence of two enzymes might explain the very efficient production of EPA in *Phaeodactylum*.

The lipids bearing the FAs that serve as substrates for E Δ6FAD, ω3FAD, and ERΔ5FAD are currently debated. Analyses have been performed in another Chromalveolate, *Monodus subterraneus*, which is not a diatom but a eustigmatophyte containing a secondary plastid. In this model, 18:1^Δ9^-precursors are mainly linked to the *sn*-2 position of PC, where it serves as a substrate for FAD2 and ERΔ6FAD. The 18:3^Δ6,9,12^ is then released, elongated into 20:3^Δ8,11,15^, and incorporated into PE, where it serves as a substrate for ω3FAD and ERΔ5FAD [44]. The lipids presenting the FA to the front-end desaturases are yet to be determined in *Phaedoactylum*, but possibly involve PC, PE, and DGTA. Indeed, all these lipids were shown to contain intermediate unsaturated FA, upstream EPA [10]. In Table 2, DGTA was therefore listed as a possible substrate for FA desaturation, although this hypothesis should be demonstrated experimentally.

Regardless of its synthesis route, EPA is then massively transferred to plastid glycerolipids, with EPA detected in 20%–50% of MGDG or DGDG molecular species, and >50% of acyl-SQDG [10]. Some Chromalveolata like *Chromera velia* accumulate ~80% EPA in MGDG and DGDG [45]. The mechanism transferring EPA to galactolipids, called the “omega pathway” by Petroutsos *et al.* [8], is currently uncharacterized.

#### 2.4.13. The (22:5^Δ7,10,13,16,19^ → 22:6^Δ4,10,13,16,19^)-desaturation by a Δ4 Front-End desaturase, ERΔ4FAD

In *Phaedoactylum*, low amounts of docosahexaenoic acid (DHA), 22:6^Δ4,10,13,16,19^, can be synthesized, and can be found in such lipids as PE or DGTA [10,46]. DHA is generated following EPA elongation into 22:5^Δ7,10,13,16,19^ catalyzed by a Δ5-elongase, and its subsequent desaturation by a Δ4FAD. Using a Δ4FAD enzyme from *Thalassiosira* as a template, we mined the *Phaeodactylum* genome and found a putative Δ4FAD (Phatr_22510) that might be responsible for desaturation of 22:5^Δ7,10,13,16,19^ into 22:6^Δ4,10,13,16,19^. By heterologous expression of an additional Δ5-elongase from *Ostreococcus* in *Phaeodactylum*, it was shown that the production of DHA could be strikingly increased, thus showing that the endogenous Δ5-elongase was limiting, whereas that of the Δ4FAD was not [46]. Like for EPA synthesis, the lipid harboring the substrate for the desaturation by the Δ4FAD enzyme has to be determined. DHA is a minor FA in *Phaeodactylum* and might reflect a different role compared to EPA.

### 2.5. Brief Overview of the Roles of Desaturases in Phaeodactylum Tricornutum

**Figure 4:**
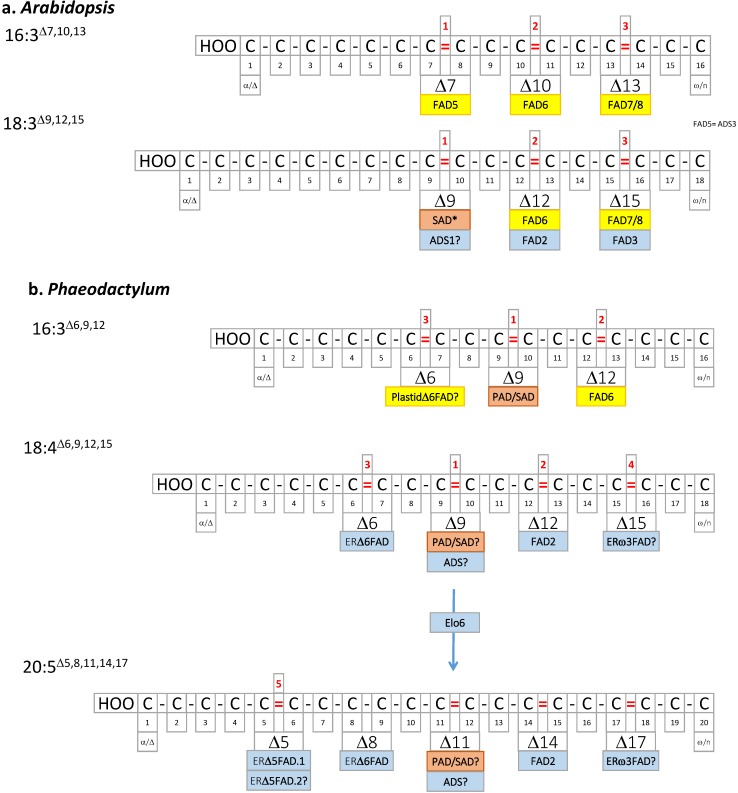
(**a**) *Arabidopsis* PUFAs; (**b**) *Phaeodactylum* PUFAs. Double bonds are localized based on the Δ nomenclature. Desaturases catalyzing the introduction of the corresponding double bond are shown in pink (stroma of chloroplast enzyme), yellow (chloroplast membrane enzymes), or light blue (ER membrane enzymes). Elo6, elongase.

Figure 4 summarizes the origin of double bonds in *Arabidopsis* and *Phaeodactylum* PUFAs, also showing the relative order of action of each enzyme (numbered 1, 2, *etc.*). FAs are not randomly desaturated. A very high level of control is exerted, highlighting even further that unsaturated FAs are likely to play specific functions.

The role of desaturases in a given organism is a difficult question. The general variations of the levels of PUFA and more specifically of EPA in *Phaeodactylum* have been reviewed recently [13]. Some physiological functions can be attributed based on physicochemical properties. It is thus commonly considered that adding double bonds to a FA improves the lateral fluidity of the harboring lipid within the membranes, and therefore the tolerance to temperature changes. The optimum temperature for the growth of *Phaeodactylum* is ~20 °C. EPAs and PUFAs increase significantly in *Phaeodactylum* grown at 10 °C [47]. The physicochemical properties of VLC-PUFAs were recently shown to be critical for the flexibility and curvature of membranes [48]. Such a role in *Phaeodactylum* or any photosynthetic organism has not, to our knowledge, been investigated yet.

Light also impacts on the level of desaturated FA, as shown by the effect of high light triggering a decrease of EPA in *Phaeodactylum* [13]. This might be consistent with the higher sensitivity of PUFAs to oxidation. Again, the presence of VLC-PUFAs in chloroplast lipids, at the most critical location for oxidative stresses in a photosynthetic cell, is puzzling. The specific roles of EPA in *Phaeodactylum* galactolipids and acyl-SQDG should therefore be analyzed in depth.

Chemical composition was shown to possibly impact on the proportion of PUFAs in *Phaeodactylum*, like an increase of silicate apparently triggering a decrease of EPA [49]. However, this response to the availability of some elements should be taken with caution when analyzing FA total content, firstly because the opposite trend was observed in other diatoms exposed to various silica levels [13], and secondly because total FAs also comprise FAs esterified to the triacylglycerol molecules that accumulate upon a nutrient stress, which contain lower levels of EPA [10]. Effects of environmental changes should therefore take into consideration the level of EPA in each lipid class, rather than the proportion of EPA in the total FA profile.

Other possible roles known for PUFAs include more refined metabolic functions, like their utilization as precursors for oxygenated forms called oxylipins, including diatom specific polyunsaturated aldehydes [50]. *Phaeodactylum* does not contain any lipoxygenase and it is still debated whether this species could produce some oxylipins from its PUFAs, acting as cell-to-cell signaling compounds. The specific toxicity of EPA produced by *Phaeodactylum* against bacteria has also been described [51], but the ecological importance of this phenomenon should be evaluated.

No specific functional characterization has currently been made to attempt to assign more specific roles to each desaturase in *Phaeodactylum*. For instance, FAD4 has been shown in other photosynthetic organisms to add a *trans*-double bond onto 16:0 esterified at position *sn-*2 of PG, generating a 16:*trans*-1^Δ3^-PG form that binds to photosystems. It is reasonable to speculate that FAD4 plays the same role in *Phaeodactylum*. As mentioned above, *Arabidopsis* FAD5 or *Chlamydomonas* CrΔ4FAD desaturases can add a double bond on a FA of MGDG, which prevents its subsequent conversion into DGDG. Therefore, these desaturases have a function in the very fine tuning of the MGDG/DGDG balance within the photosynthetic membranes. Could FAD6 and the Plastidω3FAD play a similar role in *Phaeodactylum*? Eventually the remarkably high level of EPA in galactolipids might be related to a specific molecular or biophysical property of this VLC-PUFA: a clear challenge is then to comprehend the function of EPA in MGDG, DGDG, and acyl-SQDG.

## 3. Experimental Section

### 3.1. Retrieval of Desaturase Candidate Gene Sequences

All sequences listed in this work have been retrieved from the Joint Genome Institute [52] and gene IDs were given as Phatdraft accessions, according to the ongoing structural annotation of genes models of *Phaeodactylum tricornutum* Pt1 8.6 [53]. When characterized in previous works, Phatdraft accessions of corresponding genes were simply obtained and provided in this article for consistency. For partly annotated or unannotated genes, sequences were retrieved based on BLASTP searches [54] using *Arabidopsis*, *Chlamydomonas*, or *Thalassiosira* desaturase gene models, as described in the text. Retrieved open reading frame sequences were then examined manually to discard fragments and determine full length sequences, based on the presence of an initial methionine, a STOP codon, an alignment with known ESTs, and consistency of multiple alignments [55] with gene homologues from other photosynthetic eukaryotes. The presence of consensus regions and domains characterizing soluble or membrane desaturases, as well as the detection cytochrome b5 fusions, was checked using Pfam hidden Markov models [56] annotated in InterPro [57] and checked manually.

### 3.2. Prediction of Subcellular Localization

There is no tool specifically developed for the prediction of the subcellular localization of protein sequences in *Phaeodactylum*, and most notably for the 4-membrane chloroplast. The presence of a motif frequently detected in bipartite plastid transit peptides of heterokonts, called ASAFAP, can be detected by scanning the *N*-terminal sequence with a logo profile [37,38]. In *Phaeodactylum* sequences, the core alanine-phenylalanine dipeptide (AF) was therefore detected using ASAFind [37,58] and HECTAR [38,59] online tools, as well as serine residues upstream and downstream of the AF dipeptide. The prediction of the localization in plastids was consolidated by the combined presence of a signal peptide (Sp), supporting the notion that protein precursors might reach the outermost membrane of the chloroplast connected to the ER, and a chloroplast-like transit peptide (Ctp), supporting the idea that protein precursors might go across the innermost membranes of the chloroplast. These features at the terminal end of protein sequences were predicted using generic tools developed for eukaryotes, *i.e.*, SignalP and ChloroP [60,61].

## 4. Conclusions

In this work, we mined the complete genome of *Phaeodactylum* with gene templates from *Arabidopsis*, *Chlamydomonas*, *Thalassiosira*, and other photosynthetic organisms in an attempt to list the most complete census of fatty acid desaturases. Putative and characterized *Phaeodactylum* desaturase sequences are provided as a supplementary file (Supplementary Information), and were used to predict subcellular localization in broad terms, *i.e.*, chloroplastic *vs.* cytosolic (Figure 2 and Figure 4). Substrate specificity was tentatively assessed and provided in Table 2. One important desaturase seems to be missing, *i.e.*, the ERω3FAD involved in the EPA pathway. The possible involvement of the Plastidω3FAD/FAD7 or of a cytosolic isoform of this protein should be investigated. Besides FAD2, FAD6, ERD6FAD, and ERD5FAD, which have been investigated *in vitro*, in heterologous systems, and by genetic engineering in transformed *Phaeodactylum* cells, future works must now target the characterization of other enzymes and associated proteins. In particular, an important challenge lies in the comprehension of the coordination of desaturases and elongases in the very efficient production of EPA, and the entry of this VLC-PUFA into the omega pathway that leads to its striking accumulation in chloroplast glycerolipids.

## Supplementary Files

Supplementary File 1
