# Correction: Dolch, L.-J. and Maréchal, E. Inventory of Fatty Acid Desaturases in the Pennate Diatom *Phaeodactylum tricornutum*. *Mar. Drugs* 2015, *13*, 1317–1339

**DOI:** 10.3390/md13095732

**Published:** 2015-09-09

**Authors:** Lina-Juana Dolch, Eric Maréchal

**Affiliations:** Laboratory of Plant and Cell Physiology/Laboratoire de Physiologie Cellulaire et Végétale, Unité mixte de recherche 5168 CNRS-CEA-Université Grenoble Alpes, Institut de Recherche en Sciences et Technologies pour le Vivant, CEA Grenoble, 17 rue des Martyrs, 38054 Grenoble Cedex 9, France; E-Mail: lina-juana.dolch@cea.fr

The authors wish to make the following corrections to this paper [1]:

We have found eight inadvertent errors in our paper published in *Mar. Drugs* [1]. 

On page 1327, Line 6 and column 9 of Table 2, “18:3^Δ6,12,15^” should be “18:3^Δ6,9,12^”. Line 8 and column 8 of Table 2, “20:4^Δ5,8,11,14,17^” should be “20:4^Δ8,11,14,17^”. Line 9 and column 7, “Δ5/*cis*” should be “Δ4/*cis*”. Therefore replace this incorrect table:

**Table 2:** Fatty acid desaturases of *Phaeodactylum tricornutum—*Characterized or predicted localization and substrate specificity. (*) functionally characterized; (?) based on predictions.

Name	Gene ID	Subcellular Localization	Main Substrate					Main Product
			Acyl Linked to:	Carbon Number	Presence of Double Bonds	Position and Configuration of the Introduced Double Bond	Overall Structure	Overall Structure
PAD/SAD	Phatraft_9316	Chloroplast stroma	ACP	16 and 18	0	Δ9/*cis*	16:0-ACP	16:1^Δ9^-ACP
ADS	Phatr_28797	Endomembrane system	CoA	18?	0	Δ9/*cis*?	18:0-CoA?	18:1^Δ9^-CoA?
FAD2 (*)	Phatr_25769	ER	Phospholipid/ Betaine lipid?	18	1	Δ12 (or ω6)/*cis*	18:1^Δ9^-PL (-BL?)	18:2^Δ9,12^-PL (-BL?)
ERΔ6FAD (*)	Phatr_2948	ER	Phospholipid/ Betaine lipid?	18	2	Δ6/*cis*	18:2^Δ9,12^-PL (-BL?)	18:3^Δ6,12,15^-PL (-BL?)
ERω3FAD (?)	?	ER	Phospholipid/ Betaine lipid?	18	3	Δ15 (or ω3)/*cis*	18:3^Δ6,9,12^-PL (-BL?)	18:4^Δ6,9,12,15^-PL (-BL?)
ERΔ5FAD.1 (*) ERΔ5FAD.2 (?)	Phatr_46830 Phatr_22459	ER	Phospholipid/ Betaine lipid?	20	4	Δ5/*cis*	20:4^Δ5,8,11,14,17^-PL (-BL?)	20:5^Δ5,8,11,14,17^-PL (-BL?)
ERΔ4FAD	Phatr_22510?	ER	Phospholipid/ Betaine lipid?	22	5	Δ5/*cis*	22:5^Δ7,10,13,16,19^-PL (-BL?)	22:6^Δ4,7,10,13,16,19^-PL (-BL?)
FAD6 (*)	Phatr_48423	Chloroplast membranes	*sn*1/*sn*2-MGDG/DGDG + SQDG	16	1	Δ12/*cis*	16:1^Δ9^-*sn*2-MGDG/DGDG/SQDG	16:2^Δ9,12^-*sn*1/*sn*2-MGDG/DGDG/SQDG
PlastidΔ6FAD (?)	Phatr_50443	Chloroplast membranes	*sn*2-MGDG	16	2	Δ6/*cis*	16:2^Δ9,12^-*sn*2-MGDG	16:3^Δ6,9,12^-*sn*2-MGDG
Plastidω3FAD/FAD7 (?)	Phatr_41570	Chloroplast membranes	*sn*2-MGDG?	16	3	ω3/*cis*	16:3^Δ6,9,12^-*sn*2-MGDG	16:4^Δ6,9,12,15^-*sn*2-MGDG
FAD4	Phatr_41301	Chloroplast membranes	*sn*2-PG	16	0	Δ3/*trans*	16:0-*sn*2-PG	16:1^Δ3trans^-*sn*2-PG

With the following corrected table:

**Table 2:** Fatty acid desaturases of *Phaeodactylum tricornutum—*Characterized or predicted localization and substrate specificity. (*) functionally characterized; (?) based on predictions.

Name	Gene ID	Subcellular Localization	Main Substrate					Main Product
			Acyl Linked to:	Carbon Number	Presence of Double Bonds	Position and Configuration of the Introduced Double Bond	Overall Structure	Overall Structure
PAD/SAD	Phatraft_9316	Chloroplast stroma	ACP	16 and 18	0	Δ9/*cis*	16:0-ACP	16:1^Δ9^-ACP
ADS	Phatr_28797	Endomembrane system	CoA	18?	0	Δ9/*cis*?	18:0-CoA?	18:1^Δ9^-CoA?
FAD2 (*)	Phatr_25769	ER	Phospholipid/ Betaine lipid?	18	1	Δ12 (or ω6)/*cis*	18:1^Δ9^-PL (-BL?)	18:2^Δ9,12^-PL (-BL?)
ERΔ6FAD (*)	Phatr_2948	ER	Phospholipid/ Betaine lipid?	18	2	Δ6/*cis*	18:2^Δ9,12^-PL (-BL?)	18:3^Δ6,9,12^-PL (-BL?)
ERω3FAD (?)	?	ER	Phospholipid/ Betaine lipid?	18	3	Δ15 (or ω3)/*cis*	18:3^Δ6,9,12^-PL (-BL?)	18:4^Δ6,9,12,15^-PL (-BL?)
ERΔ5FAD.1 (*) ERΔ5FAD.2 (?)	Phatr_46830 Phatr_22459	ER	Phospholipid/ Betaine lipid?	20	4	Δ5/*cis*	20:4^Δ8,11,14,17^-PL (-BL?)	20:5^Δ5,8,11,14,17^-PL (-BL?)
ERΔ4FAD	Phatr_22510?	ER	Phospholipid/ Betaine lipid?	22	5	Δ4/*cis*	22:5^Δ7,10,13,16,19^-PL (-BL?)	22:6^Δ4,7,10,13,16,19^-PL (-BL?)
FAD6 (*)	Phatr_48423	Chloroplast membranes	*sn*1/*sn*2-MGDG/DGDG + SQDG	16	1	Δ12/*cis*	16:1^Δ9^-*sn*2-MGDG/DGDG/SQDG	16:2^Δ9,12^-*sn*1/*sn*2-MGDG/DGDG/SQDG
PlastidΔ6FAD (?)	Phatr_50443	Chloroplast membranes	*sn*2-MGDG	16	2	Δ6/*cis*	16:2^Δ9,12^-*sn*2-MGDG	16:3^Δ6,9,12^-*sn*2-MGDG
Plastidω3FAD/FAD7 (?)	Phatr_41570	Chloroplast membranes	*sn*2-MGDG?	16	3	ω3/*cis*	16:3^Δ6,9,12^-*sn*2-MGDG	16:4^Δ6,9,12,15^-*sn*2-MGDG
FAD4	Phatr_41301	Chloroplast membranes	*sn*2-PG	16	0	Δ3/*trans*	16:0-*sn*2-PG	16:1^Δ3trans^-*sn*2-PG

On page 1330, line 9, “18:3 and 14” should be “18:3 and 18:4”. Line 29, in the title of Section 2.4.10, “The (18:2^Δ9,12^ → 18:3^Δ6,9,12^ and 18:3^Δ9,12,15^ → 18:3^Δ6,9,12,15^)-Desaturation” should be “The (18:2^Δ9,12^ → 18:3^Δ6,9,12^ and 18:3^Δ9,12,15^ → 18:4^Δ6,9,12,15^)-Desaturation”. On page 1331, line 6, in the title of Section 2.4.11, “The Question of the (18:2^Δ9,12^ → 18:3^Δ6,9,15^ and 18:3^Δ6,9,12^ → 18:4^Δ6,9,12,15^)-Desaturation” should be “The Question of the (18:2^Δ9,12^ → 18:3^Δ9,12,15^ and 18:3^Δ6,9,12^ → 18:4^Δ6,9,12,15^)-Desaturation”. On page 1332, line 1, “E Δ6FAD” should be “ERΔ6FAD”. On page 1335, lines 25–26, in the Conclusions Section, “ERD6FAD, and ERD5FAD” should be “ERΔ6FAD, and ERΔ5FAD”.

These changes have no material impact on the conclusions of our paper. We apologize to our readers for any inconvenience caused.
