# Supplementary material for: Inventory of Fatty Acid Desaturases in the Pennate Diatom Phaeodactylum tricornutum
Source: Mar Drugs. 2015 Mar 16;13(3):1317–39. doi: 10.3390/md13031317 (PMC4377986; doi:10.3390/md13031317)
Supplement: Supplementary File 1 [file marinedrugs-13-01317-s001.pdf]

Supplementary Information

1. PLASTID Desaturases

The alanine-phenylalanine dipeptide conserved in the ASAFAP motif of heterokont plastid bipartite targeting signal is highlighted in red. Serine residues known to be enriched in the −12/+12 flanking region of the ASAFAP motif are shown in green.

PAD (Phatdr_9316)

MLSTKLFWTSSVLASSAVLAFPTSPATRTPRSTSILKVAVDPTTVTKKEYEDICGVSFDADT
LEDRLKATNYLYPKHVEVIDDIAPIAGAMVDEILLETGENAWQPQDYLPDLSQDNWHDSIKE
VRAMAKEIPDELLVVLIGDMVTEEALPTYQTLLNTFEGCDDPTGTSESPWARWSRGWTSEEN
RHGDLLNKYLYLGGRCDMRNIEVTIQHLITNGFNPQARKDPYRGFVYTSFQERATKISHGNVG
KLARTYGEKNLNKICAKIAGDEGRHEKAYQIFSEEILKRDPDGLIHVFGDMMRGQIVMPAEQ
MTDGKDPDLYDNFSMVAQKTGVYTALDYAEIIDHLVKRWDLEHLEGLSPAAEKEREYLCRL
PERYRKLATRSMNKKKKVTEDEDPLKSFGWIYGRMA

FAD6 (Phatdr_48423)

MVRFSTAALFSLSTLTPSCIGAFQLSSPAQLPTSRLRRHTNTAPLSAVAVDSGSSDPALVGN
LPLPNNNDNEDKNRRMPMMDLKGIALSGLKGQALSVRAEDFPQAKDLRAVIPKDCFEPDTAK
SLGYLSVSTMGTILCSVVGANLLSVLDPSNPLTWPLWAAYGAVTGTVAMGLWVLAHECGHG
AFSKNRSLQDAVGYIIHSIMLVPYFSWQRSHAVHHQYTNHMELGETHVPDRADKEGEKSLAL
RQFMLDSFGKDKGMKAYGGLQSFLHLIVGWPAYLLIGATGGPDRGMTNHFYPNPLSTPTQPK
KELFPGNWKEKVYQSDIGIAAVVGALIAWTVTSGLAPVMALYGGPLIVINAWLVLYTWLQHT
DTDVPHFSSDNHNFVKGALHTIDRPYDKLDPWGIIDFLHHKIGTTHVAHHFDSTIPHYKAQIAT
DAIKAKFPEVYLYDPTPIPQAMWRVAKGCTAVEQRGDAWVWKNEGIEDLVEHRQSKLSSE

PlastidDelta6FAD (Phatdr_50443)

MKFLHSALIVLTSASASSAFTATNFFCLSQYGSSVSGCPEDFIRQWKAAGSTTNRISKKNVA
YDCDEDANCVIVDACDDEQCRTSLDVRIHGKWYDLSGWRKAHPAGAHWIDWYDGRDATEV
MDAFHSEKGRAMYKRLPASSTESVAMLETTIAPDSSTQIAFRQLRDDLEKEGWWKRDMVHE
FTQLGIWASLVVGAAVTAHSAPPLATFLLGLSMTAAGWLGHDFIHGVDSFTDRLRNFAGVAA
GLGPTWWSDKHNKHHALTNEQGVDEDIATDPFLFTWAPDPKDDSPLRKIQHLIFWVPFSALF
ALWRVDTMQVVIEAVENKRVGAKGELYGLLLHYAVLFTVFPVTVWLPAIFLSGLMSALIVTP
THQSEEMFETYQPDWVTAQFQSTRNAVTTNPFSEWLWGGMQYQLEHHLFPSMPRNRYPALR
ERLIQFAADNKIPGGYRESGEFEILRMNWNLYKSVAEADAVPGAPPTRGRLGQQGAIRET
NSPAAQQEKAKIDQTVAKGNGPALESV

FAD4 (Phatdr_41301)

MILHGKTYTSCYPESSRLCSNVSKAFNKMSLPRQLAATVCFLLAAKTSSFSLSSSTRTVHRE
SGLKPLHMAVIPDPSSSSQGLYSTSLRATALASTETETTASNKEKETKKPSWNDDGFVFGLEGS
GLERPKGRNAQIVVEGDSLETQPFQVAAVSVTFAAHAGFLVNSFSGMVEASSGNIALTSIHAIV

LTLVSWVIADFGSGVLHWATDNYGNGKTPVMGGIIAAFQGHHSAPWTIAQRGFCNNVYKLCI
PFGIVPMLAINAIAPPDVVFFMATFCVFEIMSQEFHKWSHQLKSETPGWVNWLQDSGLTIARK
PHALHHLAPFEGNYCIISGICNPVLDQSGFFRRLERVVYSLNGIESNAWKLDPALRERTLSGDY
KFPKARSSSSKP

Putative PlastidOmega3FAD (Phatdr_41570)

MKLHIAPPLIISAYVFSVSIFHNTVNAFSLRIPSTHRTVFLPQVTLNAKRWMVATGVETNAA
VATPENDEIHPRRDWTHDEPPKLSEVKRMLPQEAFHIDTATSLFYFAVDFIAVASTMGFLNSV
VSSDIYLSFPIWGKFLAVAPLQILTGFAMWCMWCIGHDAGHTTVSKDRRFGALINRVVGEVA
HSAICLTPFVPWAKSHLKHHMGHNHLTRDYSHQWFIREERESLHPLIQLSHATRNLQLPILYLV
YLLFGVPDGGHVVFYGRMWEQSTAKEKADAAVSVIVSLVTAGSLWINMGLANFFVVCMVP
WLVLSFWLFMVTYLQHHSDDGLLYTDETWSFERGAFQTVDRDYGTWINRMSHHMMDGHLV
HHLFFTRVPHYRLEEATKSLYAVMAARGQSHLIKTIDTPDFTQEIAKQFDKNWFFVNENQIVRK

2. Cytosol/ER Desaturases

ADS (Phatdr_28797)

MDLSSEMLREIPGTLSQHYKAGNLNYPMIVYTTIVHTVALVGLCTIPQASAETLLWAFVLW
PISGFGITVGVHRLWSHRSYEAALPVRFVLMLCNSIANQGSIYHWSRDHRVHHKFSETDADPH
NARRGFFFAHMGWLFVKKDPAVIEAGREMDFSDLLEDPVVALQKMVDPWFALYMCYVMPA
QVASYFWGENFWTAFLVAGGLRYCFVLHCTWLVNSAAHLYGDHPYDLTSYPAENPFVSWC
AVGEGWHNWHHKYPFDYAASEFGVSSQYNPSKLVIDVLASVGLVWGRKRGTAAWAMGRA
RRDRDIAQGKEMPKQPPRPWEVRTVARKIA

FAD2 (Phatdr_25769)

MGKGGQRAVAPKSATSSTGSATLSQSKEQVWTSSYNPLAKDAPELPTKGQIKAVIPKECFQ
RSAFWSTFYLMRDLAMAAAFCYGTSQVLSTDLPQDATLILPWALGWGVYAFWMGTILTGPW
VVAHECGHGAYSDSQTFNDVVGFIVHQALLVPYFAWQYTHAKHHRRTNHLVDGESHVPSTA
KDNGLGPHNERNSFYAAWHEAMGDGAFAVFQVWSHLFVGWPLYLAGLASTGKLAHEGWW
LEERNAIADHFRPSSPMFPAKIRAKIALSSATELAVLAGLLYVGTQVGHLPVLLWYWGPYTFV
NAWLVLYTWLQHTDPSIPHYGEGEWTWVKGALSTIDRDYGIFDFFHHTIGSTHVVHHLFHEM
PWYNAGIATQKVKEFLEPQGLYNYDPTPWYKAMWRIARTCHYVESNEGVQYFKSMENVPLT
KDVRSKAA

ERDelta6FAD (PtD6) (Phatdr_2948)

MGKGGDARASKGSTAARKISWQEVKTHASPEDAWIIHSNKVYDVSNWHEHPGGAVIFTH
AGDDMTDIFAAFHAPGSQSLMKKFYIGELLPETTGKEPQQIAFEKGYRDLRSKLIMMGMFKSN
KWFYVYKCLSNMAIWAAACALVFYSDRFWVHLASAVMLGTFFQQSGWLAHDFLHHQVFTK
RKHGDLGGLFWGNLMQGYSVQWWKNKHNGHHAVPNLHCSSAVAQDGDPDIDTMPLLAWS
VQQAQSYRELQADGKDSGLVKFMIRNQSYFYFPILLLARLSWLNESFKCAFGLGAASENAAL

ELKAKGLQYPLLEKAGILLHYAWMLTVSSGFGRFSFAYTAFYFLTATASCGFLLAIVFGLGHN
GMATYNADARPDFWKLQVTTTRNVTGGHGFPQAFVDWFCGGLQYQVDHHLFPSLPRHNLA
KTHALVESFCKEWGVQYHEADLVDGTMEVLHHLGSVAGEFVVDFVRDGPAM

ERDelta5FAD1 (PtD5) (Phatdr_46830)

MAPDADKLRQRQTTAVAKHNAATISTQERLCSLSSLNGEEVCIDGIIYDLQSFDHPGGETIK
MFGGNDVTVQYKMIHPYHTEKHLEKMKRVGKVTDFVCEYKFDTGFEREIKREVFKIVRRGK
DFGTLGWFFRAFCYIAIFFYLQYHWVTTGTSWLLAVAYGVSQAMIGMNVQHDANHGATSKR
PWVNDMLGLGADFIGGSKWLWQEQHWTHHAYTNHAEMDPDSFGAEPMLLFNDYPLDHPA
RTWLHRFQAVFYMPVLAGYWLSAVFNPQILDLQQRGALSVGIRLDNAFIHSRRKYAVFWRA
VYIAVNVIAPFYTNSGLEWSWRVFGNIMLMGVAESLALAVLFSLSHNFESADRDPTAPLKKT
GEPVDWFKTQVETSCTYGGFLSGCFTGGLNFQVEHHLFPRMSSAWYPYIAPKVREICAKHGV
HYAYYPWIHQNFLSTVRYMHAAGTGANWRQMARENPLTGRA

ERDelta5FAD2 (Phatdr_22459)

MDVSLRNKSLSVDTLAPNHVCIDGKVFDLDSFDHPGGDSIHVFGGNDVTVLYKMIHPHHG
PNQYAQKMKLVGVIDKYRCEYSFDSDFGKEMKREVFQIVRRGQEFGTVGYFFRAFLYIAFFV
AVVYRWTFQTGPSYALAVVFGLAKALIGLNVQHDANHGAAAPPGRKNVWINDLLGWGADL
IGGCKYLWIQKHWTHHAYTNHAEKDPDAFAAEPFLIFREYPASHPARQWYHKYQTLLFLPIIA
GYWLSSVLSLEVAKLQDAGAMSATMKFENNFVARQRKFTVFWRIVHLVIILGPPLRQHGLTA
TALGQALTVGAAGSLFLGCLFSLSHNFVNAERDPTAILAPPPTSTDGSDNENTTAPVCWYKAQ
VETSCTYGGFVSGALTGGLNFQVEHHLFPRMSSAWYPFIAPTVRRVCAKHNVTYTYYPWLW
QNMASMMRYLHVTGGNTDAITKLE

Putative ERDelat4FAD (Phatdr_22510)

MATTTPSQSKTLDPLLLWMIHGNYYDLHTYVSRHPGGKEAILLGRGRDCTALFESYHPFTS
QHRRVLEKHRVTIDTSFCSKQQDRKVSKQTVSSSEQASDVFYNMLCQRVAHALQSQGVDPIR
DRGATWTRSIYYVFLFAALLASGYAHCTGSLLGSLLFGVAGWFIGALGHDGGHFAVSRRAW
LNDFSVWGISWLCNPIMWQHQHTYAHHSFTNEFDHDPDLHHFTTFLRVHRKFQQNCIYRNQA
NWVYVFWAYTFVTFGACFWIPWGVLREQTLYGLVDWTDRKRPSRTAAFVFHLVTYFGLVM
VLPFWTHGTWYKACLGVILHMTTSGLIFAFFSQINHINELSLDEKVVKLHRSTLDPTVRDSWA
VAQVEASNNFCTDSAFWHLFSNGLNLQIEHHLFPGINHCHLHHIAPVVRETCNEYGVRYKSY
DSWSDIMRAMLKWLDQLSVGLD

© 2015 by the authors; licensee MDPI, Basel, Switzerland. This article is an open access article distributed under the terms and conditions of the Creative Commons Attribution license (http://creativecommons.org/licenses/by/4.0/).
